# Mode of action-specific and cause-specific retention of biologic and targeted synthetic disease-modifying antirheumatic drugs in anti-SS-A antibody-positive rheumatoid arthritis: The ANSWER cohort study

**DOI:** 10.1371/journal.pone.0344747

**Published:** 2026-03-18

**Authors:** Kazuma Nishisaka, Takaichi Okano, Takumi Imai, Masanori Tsubosaka, Tomoyuki Kamenaga, Naoki Nakano, Shinya Hayashi, Wataru Yamamoto, Akira Onishi, Kosaku Murakami, Kohei Tsujimoto, Masao Katsushima, Ayaka Yoshikawa, Takuya Kotani, Hideki Amuro, Yonsu Son, Tetsu Itami, Yuji Nozaki, Yoko Nose, Mai Yamashita, Iku Shirasugi, Hirotaka Yamada, Keisuke Nishimura, Yo Ueda, Sho Sendo, Motomu Hashimoto, Ryosuke Kuroda, Jun Saegusa

**Affiliations:** 1 Department of Rheumatology and Clinical Immunology, Kobe University Graduate School of Medicine, Kobe, Japan; 2 Clinical and Translational Research Center, Kobe University Hospital, Kobe, Japan; 3 Department of Orthopaedic Surgery, Kobe University Graduate School of Medicine, Kobe, Japan; 4 Department of Health Information Management, Kurashiki Sweet Hospital, Okayama, Japan; 5 Department of Advanced Medicine for Rheumatic Diseases, Graduate School of Medicine, Kyoto University, Kyoto, Japan; 6 Center for Cancer Immunotherapy and Immunobiology, Graduate School of Medicine, Kyoto University, Kyoto, Japan; 7 Department of Respiratory Medicine and Clinical Immunology, Graduate School of Medicine, The University of Osaka, Osaka, Japan; 8 Department of Clinical Immunology, Graduate School of Medicine, Osaka Metropolitan University, Osaka, Japan; 9 Department of Internal Medicine (IV), Division of Rheumatology, Osaka Medical and Pharmaceutical University, Osaka, Japan; 10 First Department of Internal Medicine, Kansai Medical University, Osaka, Japan; 11 Department of Hematology and Rheumatology, Kindai University School of Medicine, Osaka, Japan; University of Toronto Centre for Health Promotion: University of Toronto Dalla Lana School of Public Health, CANADA

## Abstract

Anti-SS-A (Ro) antibody-positive rheumatoid arthritis (RA) constitutes a clinically important subgroup, but its impact on retention of biologic or targeted synthetic disease-modifying antirheumatic drugs (b/tsDMARDs) across different modes of action (MOA) and reasons for discontinuation remains unclear. We conducted a multicenter retrospective analysis of the Japanese ANSWER cohort, including RA patients who started or switched b/tsDMARDs between 2011 and 2024 and had baseline anti-SS-A antibody testing. Among 1,452 patients (2,703 treatment courses), 255 patients (17.6%) were anti–SS-A antibody positive (507 courses, 18.8%). Propensity score matching balanced baseline characteristics, and drug retention was evaluated using Kaplan–Meier and competing risk analyses. Overall discontinuation was analyzed using Cox proportional hazards models, and Fine–Gray subdistribution hazards models were used for discontinuation by reason and for adverse event–related discontinuation stratified by MOA. After matching, 507 treatment courses from 255 anti-SS-A antibody-positive patients and 1,014 courses from 628 antibody-negative patients were analyzed. Anti-SS-A antibody positivity was not associated with overall b/tsDMARD retention (hazard ratio [HR] 1.07, 95% confidence interval [CI] 0.91–1.26, p = 0.382). In MOA-stratified analyses, positivity showed a trend toward increased discontinuation with interleukin-6 (IL-6) receptor inhibitors and cytotoxic T lymphocyte-associated antigen 4-immunoglobulin (CTLA4-Ig). In competing-risk analyses, discontinuation due to adverse events was significantly more frequent in antibody-positive patients (subdistribution hazard ratio [sHR] 1.80, 95% CI 1.28–2.52; p = 0.000685). Among adverse event-related discontinuations, anti-SS-A antibody positivity was associated with higher risks with IL-6 receptor inhibitors (sHR 2.41, 95% CI 1.24–4.71; p = 0.0098) and tumor necrosis factor (TNF) inhibitors (sHR 2.04, 95% CI 1.22–3.40; p = 0.0066), but not with CTLA4-Ig or Janus kinase (JAK) inhibitors. These findings suggest that treatment tolerability, rather than overall efficacy, may be a key determinant of b/tsDMARD survival in anti-SS-A antibody-positive RA and that MOA- and cause-specific profiles should be considered when selecting therapies in this subgroup.

## Introduction

Rheumatoid arthritis (RA) is a chronic inflammatory disease characterized by progressive joint destruction and disability [1–5]. The advent of biologic and targeted synthetic disease-modifying antirheumatic drugs (bDMARDs and tsDMARDs) has markedly improved clinical outcomes in RA, providing additional therapeutic options for patients who do not respond adequately to conventional treatments [6–9]. However, the treatment response and drug retention rates for biologic or targeted synthetic disease-modifying antirheumatic drugs (b/tsDMARDs) vary among individuals.

Anti-SS-A (Ro) antibody-positive RA constitutes a clinically important subgroup [10–12]. Approximately 20% of Japanese patients with RA are positive for anti-SS-A antibodies, with frequent overlap with Sjögren disease (SjD) [13]. Recent studies have indicated that anti-SS-A antibody-positive patients may be more difficult to treat than antibody-negative patients because of a reduced response to methotrexate monotherapy and less improvement in subjective symptoms [14]. However, the effect of anti-SS-A antibodies on the efficacy and retention of b/tsDMARDs, especially across different modes of action (MOA), has not been thoroughly investigated. Some reports suggest that tumor necrosis factor (TNF) inhibitors may be less effective in anti-SS-A antibody-positive RA [15–17], but limited data are available on retention and reasons for discontinuation of other b/tsDMARDs. To address this knowledge gap, we analyzed a large multicenter registry to compare drug retention among patients with RA with or without anti-SS-A antibodies, with a particular focus on MOA and reasons for discontinuation.

## Materials and methods

### Study design and participants

We conducted a retrospective observational study using data from the ANSWER cohort (Kansai Consortium for Well-being of Rheumatic Disease Patients), a multicenter registry in Japan. We included all patients with RA who initiated or switched to b/tsDMARDs between 2011 and 2024 and had anti-SS-A antibody testing at baseline. Patients could contribute multiple treatment courses if they initiated or switched to a different b/tsDMARD during the study period. For each treatment course, baseline was defined as the date of initiation or switching to each b/tsDMARD treatment course [18]. Baseline covariates were obtained at baseline or from the closest available measurement within 90 days prior to treatment initiation/switch date. Because this is a real-world multicenter cohort in which clinical assessments are not always recorded on the exact index date, this pre-index window was used to reduce missingness and reflect routine clinical practice, consistent with prior analyses from the ANSWER cohort [19]. Radiographic severity and functional status were assessed using the Steinbrocker stage and the global functional class (both recorded as 1–4 in this study); stage 1 (I) indicates non-erosive disease and stage 4 (IV) indicates severe destruction with bony ankylosis, while class 1 (I) indicates full ability to perform usual activities of daily living and class 4 (IV) indicates limitation in usual self-care activities. [20,21] RA was diagnosed according to the 1987 American College of Rheumatology (ACR) classification criteria [22] or the 2010 ACR/European League Against Rheumatism (EULAR) criteria [23]. Anti-SS-A antibody positivity was defined as a titer ≥10 U/mL by Enzyme-Linked Immunosorbent Assay (ELISA) or Chemiluminescent Enzyme Immunoassay (CLEIA), according to assay reference ranges used in clinical practice, or ≥1 by double immunodiffusion (DID) [24–26]. In this multicenter registry, anti–SS-A antibodies were measured using different assay platforms across institutions and over time (e.g., ELISA, CLEIA, or DID). When the assay “method” field was missing but a quantitative unit (U/mL) was recorded, we categorized these results as “Quantitative (method missing)” and applied the same positivity threshold used for quantitative assays (≥10 U/mL). To ensure data quality, the analysis was restricted to four facilities with a Clinical Disease Activity Index (CDAI) missing data rate below 30%. Because CDAI at treatment-course baseline was required for confounding adjustment, we restricted the analysis to facilities where baseline CDAI was recorded for at least 70% of treatment courses (missingness <30%). This criterion was applied to treatment-course baseline (week 0) CDAI and not to CDAI measurements during follow-up. Follow-up was defined from the date of drug initiation to the earliest of 24 months, loss to follow-up, drug discontinuation (including switching to another agent), or death.

### Exposure and outcomes

The patients were divided into two groups based on the presence or absence of anti-SS-A antibodies. The primary outcome was the overall retention of all b/tsDMARDs. Secondary outcomes included drug retention stratified by (1) mode of action (MOA) of b/tsDMARDs and (2) reason for discontinuation (ineffectiveness, adverse events, and remission). The b/tsDMARDs analyzed included TNF inhibitors (adalimumab, certolizumab pegol, etanercept, golimumab, infliximab, ozoralizumab, and their biosimilars), interleukin-6 (IL-6) receptor inhibitors (tocilizumab and sarilumab), cytotoxic T lymphocyte-associated antigen 4-immunoglobulin (CTLA4-Ig: abatacept), and Janus kinase (JAK) inhibitors (baricitinib, tofacitinib, peficitinib, upadacitinib, and filgotinib).

### Statistical analysis

Overall drug retention, including all reasons for discontinuation, was visualized using the Kaplan–Meier method. A competing risks framework was used for the analysis stratified by reason for discontinuation, utilizing cumulative incidence functions. In this framework, alternative reasons for discontinuation were treated as competing events. Cumulative incidence functions were used for visualization in the presence of competing risks, and Fine–Gray subdistribution hazards models were fitted to estimate subdistribution hazard ratios (sHRs) corresponding to the cumulative incidence functions [27,28]. As an exploratory within–TNF inhibitor analysis, motivated by reported differences in immunogenicity among TNF inhibitors [29–32], we grouped etanercept (including biosimilars), golimumab, and ozoralizumab versus other TNF inhibitors, and fitted Fine–Gray models for discontinuation due to ineffectiveness including an interaction term between anti–SS-A antibody status and TNF inhibitor subgroup. Analyses were conducted at the treatment-course level, as patients could contribute multiple treatment courses. As a complementary analysis, cause-specific hazard ratios (csHRs) for drug discontinuation were estimated using Cox proportional hazards models. Robust variance estimates clustered by matching design and patient. To adjust for potential confounding factors, propensity score matching was conducted using the following covariates: age, sex, disease duration, glucocorticoid dosage (prednisolone equivalent), methotrexate (MTX) dosage, seropositivity (rheumatoid factor [RF], anti-citrullinated peptide antibody [ACPA]), CDAI, radiographic stage, functional class, MOA of b/tsDMARDs, and the number of prior b/tsDMARDs. The registry does not systematically capture a structured diagnosis of concomitant SjD; therefore, SjD status could not be included as a covariate in the propensity score model. Matching was performed using the nearest-neighbor method in a 1:2 ratio (one anti-SS-A antibody-positive case to two anti-SS-A antibody-negative cases). Post-matching covariate balance was assessed using standardized mean differences (SMD), with values below 0.2 considered acceptable. For variables with missing data, multiple imputation by chained equations (MICE) was used. Effect estimates were obtained within each imputed dataset and pooled across the 100 imputations according to Rubin’s rules using the within approach [33]. For descriptive summaries and graphical presentation (Kaplan–Meier and cumulative incidence curves), we present results from one representative imputed matched dataset (imputation 1). To address potential heterogeneity in anti–SS-A assay methods and the possibility of misclassification, we conducted sensitivity analyses by (i) excluding “Quantitative (method missing)” records, (ii) restricting analyses to quantitative assays by excluding DID results, and (iii) additionally including the assay-method category in the propensity score model. These sensitivity analyses were applied to the primary time-to-event analyses, including competing-risk models for reason-specific discontinuation. All statistical analyses were performed using R, version 4.4.3.

### Ethics statement

This multicenter observational study used data from the ANSWER cohort (Kansai Consortium for Well-being of Rheumatic Disease Patients) and was conducted in accordance with the Declaration of Helsinki and relevant national and institutional guidelines. The study protocol was reviewed and approved by the Ethics Review Committee of Osaka Metropolitan University (approval no. 2023−053; July 25, 2023) and the Ethics Committee/Institutional Review Board (IRB) of Kobe University Hospital (approval no. B232067), as well as the ethics committees of the participating institutions. Written informed consent was obtained from patients who were actively receiving care at the participating institutions. For patients who had already transferred to other hospitals or clinics, information about the study and the opportunity to opt out were provided via public postings on the participating hospitals’ websites. Data for this retrospective analysis were extracted from the ANSWER cohort database in September 17, 2024. During data extraction and analysis, the investigators had access only to de-identified data; all direct personal identifiers were removed at the data center before the dataset was provided to the authors.

## Results

### Baseline characteristics

**Fig 1:**
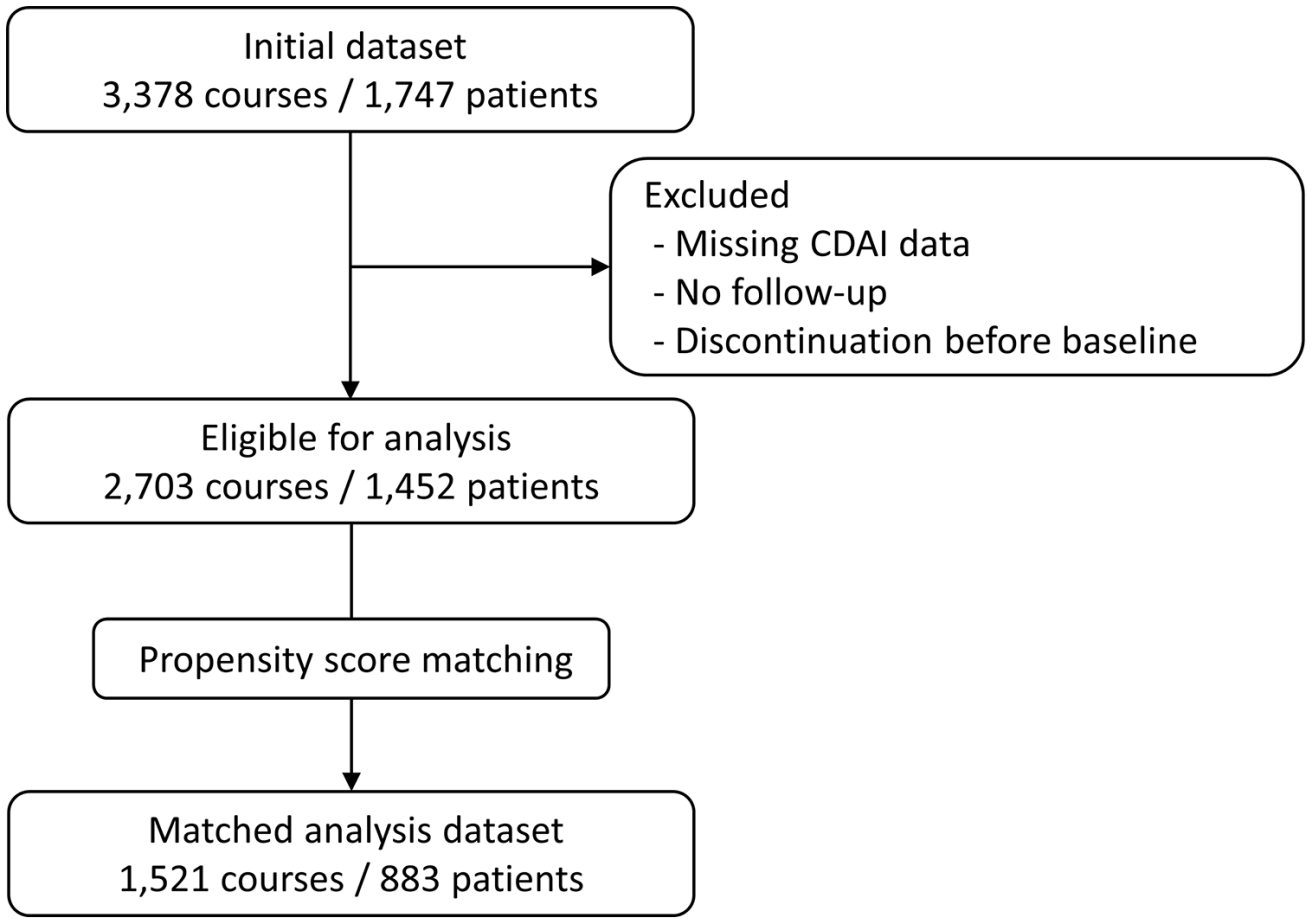
Study flow diagram. The flow chart shows the selection of treatment courses and patients included in the analysis. Of the initial 3,378 treatment courses from 1,747 patients in the ANSWER cohort, 675 courses (295 patients) were excluded due to missing Clinical Disease Activity Index (CDAI) data, lack of follow-up, or discontinuation before treatment initiation. The eligible dataset before propensity score matching included 2,703 courses from 1,452 patients. After propensity score matching, the matched analysis dataset comprised 1,521 courses from 883 patients. **Abbreviations:** CDAI, Clinical Disease Activity Index.

A total of 2,703 treatment courses from 1,452 patients were included in the analysis (Fig 1). Among these, 507 (18.8%) treatment courses were from 255 (17.6%) anti-SS-A antibody–positive patients, and 2,196 (81.2%) treatment courses were from 1,197 (82.4%) anti-SS-A antibody–negative patients. (Table 1 and S1 Table). S1 Table summarizes patient-level baseline characteristics using each patient’s first b/tsDMARD treatment course; therefore, values are presented as the number of patients. Baseline radiographic stage (Steinbrocker stage) and functional class (global functional class) are also summarized in Table 1 and S1 Table. Before matching, notable differences were observed between the groups for variables such as age, sex, seropositivity (RF, ACPA), and prior b/tsDMARD exposure, with several covariates showing SMDs greater than 0.2. After propensity score matching, the matched dataset included 507 treatment courses from 255 anti-SS-A antibody-positive patients and 1,014 courses from 628 antibody-negative patients. The covariate balance was substantially improved, with SMDs for all variables reduced to below 0.2. Among anti–SS-A antibody–positive courses, anti–SS-B and fluorescent antinuclear antibody (FANA) results were available in 361/507 (71.2%) and 406/507 (80.1%), respectively; corresponding availability at the patient level (first course) was 177/255 (69.4%) and 197/255 (77.3%). Anti–SS-A assay methods were heterogeneous across sites (ELISA, CLEIA, DID, and “Quantitative [method missing]”; see Methods), and the distribution by assay is summarized for transparency. At the treatment-course level, the distribution was ELISA (n = 1,283; of which n = 239 were positive), CLEIA (n = 794; n = 146 positive), DID (n = 518; n = 88 positive), and Quantitative (method missing) (n = 108; n = 34 positive). At the patient level (first course), the distribution was ELISA (n = 638; n = 118 positive), CLEIA (n = 424; n = 73 positive), DID (n = 319; n = 45 positive), and Quantitative (method missing) (n = 71; n = 19 positive). Among positives in the “Quantitative (method missing)” category (n = 34), titers were generally not concentrated near the cutoff (median 77.8 U/mL, IQR 32.4–1200; range 19.1–1200).

**Table 1:** Baseline characteristics of treatment courses before and after propensity score matching.

	Before Matching				After Matching			
Variable	Overall	anti-SS-A antibody		Difference	Overall	anti-SS-A antibody		Difference
		Negative	Positive			Negative	Positive	
	N = 2,703	N = 2,196	N = 507		N = 1,521	N = 1,014	N = 507	
Age (years)	59 ± 16	60 ± 16	55 ± 15	0.34	55 ± 16	55 ± 16	55 ± 15	0.00
Sex				0.57				0.06
Male	475 (18%)	459 (21%)	16 (3.2%)		59 (3.9%)	43 (4.2%)	16 (3.2%)	
Female	2,228 (82%)	1,737 (79%)	491 (97%)		1,462 (96%)	971 (96%)	491 (97%)	
Disease Duration (months)	113 ± 118	112 ± 122	117 ± 103	−0.05	112 ± 108	110 ± 110	117 ± 103	−0.07
PSL (mg/day)	2.0 ± 5.9	2.0 ± 6.3	2.1 ± 3.9	−0.01	2.1 ± 6.9	2.1 ± 8.0	2.1 ± 3.9	0.00
MTX (mg/week)	3.7 ± 5.0	3.7 ± 4.8	3.8 ± 5.8	−0.03	4.0 ± 5.3	4.0 ± 5.0	3.8 ± 5.8	0.04
RF positive	1,941 (73%)	1,525 (71%)	416 (85%)	−0.35	1,285 (84%)	852 (84%)	433 (85%)	−0.04
ACPA positive	1,947 (77%)	1,551 (75%)	396 (86%)	−0.30	1,305 (86%)	867 (86%)	438 (86%)	−0.03
b/tsDMARDs Line	2 ± 2	2 ± 2	2 ± 2	−0.16	2 ± 2	2 ± 2	2 ± 2	−0.05
CDAI	17 ± 11	17 ± 11	17 ± 12	−0.01	16 ± 11	16 ± 11	17 ± 11	−0.02
Stage				−0.10				−0.03
1	827 (37%)	693 (38%)	134 (30%)		527 (35%)	369 (36%)	158 (31%)	
2	584 (26%)	462 (25%)	122 (28%)		375 (25%)	237 (23%)	138 (27%)	
3	323 (14%)	238 (13%)	85 (19%)		241 (16%)	145 (14%)	96 (19%)	
4	529 (23%)	430 (24%)	99 (23%)		378 (25%)	263 (26%)	115 (23%)	
Class				0.12				−0.01
1	650 (28%)	520 (28%)	130 (29%)		495 (33%)	342 (34%)	153 (30%)	
2	1,181 (51%)	930 (50%)	251 (56%)		773 (51%)	494 (49%)	279 (55%)	
3	434 (19%)	376 (20%)	58 (13%)		237 (16%)	168 (17%)	69 (14%)	
4	33 (1.4%)	27 (1.5%)	6 (1.3%)		16 (1.1%)	10 (1.0%)	6 (1.2%)	
b/tsDMARDs				0.07				0.05
TNFi	1,327 (49%)	1,090 (50%)	237 (47%)		735 (48%)	498 (49%)	237 (47%)	
IL6i	620 (23%)	495 (23%)	125 (25%)		360 (24%)	235 (23%)	125 (25%)	
CTLA4-Ig	368 (14%)	300 (14%)	68 (13%)		194 (13%)	126 (12%)	68 (13%)	
JAKi	388 (14%)	311 (14%)	77 (15%)		232 (15%)	155 (15%)	77 (15%)	

Values are presented as mean ± standard deviation (SD) for continuous variables, and n (%) for categorical variables. “Difference” indicates the standardized mean difference (SMD) between anti-SS-A antibody-positive and -negative groups. “Before matching” and “After matching” refer to the datasets prior to and following propensity score matching, respectively. Baseline was defined at the initiation or switching of each biologic and targeted synthetic disease-modifying antirheumatic drug (b/tsDMARD) treatment course. Counts represent treatment courses. Propensity scores were calculated using the following covariates: age, sex, disease duration, glucocorticoid dosage, methotrexate dosage, seropositivity (RF, ACPA), CDAI, radiographic stage, functional class, mode of action (MOA) of b/tsDMARDs, and the number of prior b/tsDMARDs. Radiographic stage indicates the Steinbrocker stage (1–4), and functional class indicates the global functional class (1–4). For presentation, this table shows one representative imputed dataset (imputation 1) from the 100 multiply imputed datasets. Similar trends were observed across the other imputed datasets, with all post-matching SMDs < 0.20. **Abbreviations**: ACPA, anti-citrullinated peptide antibody; b/tsDMARD, biologic and targeted synthetic disease-modifying antirheumatic drug; CDAI, Clinical Disease Activity Index; CTLA4-Ig, cytotoxic T lymphocyte-associated antigen 4-immunoglobulin; IL6i, interleukin-6 receptor inhibitor; JAKi, Janus kinase inhibitor; MTX, methotrexate; PSL, prednisolone; RF, rheumatoid factor; TNFi, tumor necrosis factor inhibitor.

### Overall drug retention rate of b/tsDMARDs

**Fig 2:**
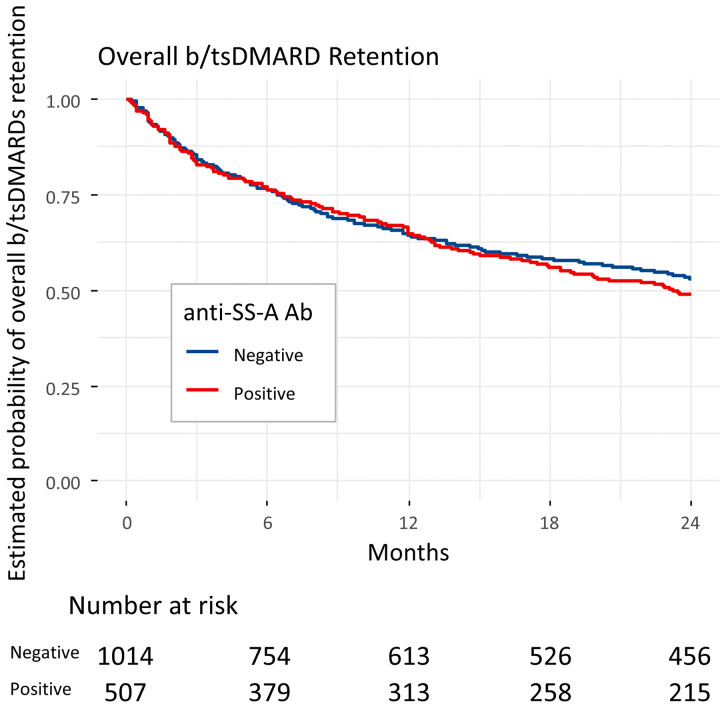
Kaplan–Meier curves of overall b/tsDMARD retention in RA patients with or without anti-SS-A antibodies. Kaplan–Meier curves show the overall retention of biologic and targeted synthetic disease-modifying antirheumatic drug (b/tsDMARD) over 24 months in rheumatoid arthritis (RA) patients according to anti-SS-A antibody status. Overall retention rates were similar between antibody-positive and -negative patients. Analyses were performed after propensity score matching. The number at risk is shown below the plot. The curves are shown for one representative imputed matched dataset (imputation 1). **Abbreviations:** Ab, antibody; b/tsDMARD, biologic and targeted synthetic disease-modifying antirheumatic drug; CI, confidence interval; HR, hazard ratio; RA, rheumatoid arthritis.

Kaplan–Meier curves demonstrated a similar pattern of overall b/tsDMARD retention between the antibody-positive and -negative groups over the two-year period (Fig 2). In Cox proportional hazards models, anti-SS-A antibody positivity was not associated with overall b/tsDMARD retention (hazard ratio [HR], 1.07; 95% confidence interval [CI]: 0.91–1.26; p = 0.382). Sensitivity analyses addressing heterogeneity in anti–SS-A assay methods (excluding “Quantitative (method missing)”, excluding DID, and additionally adjusting for assay-method category) yielded results consistent with the primary analysis.

### Drug retention by mode of action

**Fig 3:**
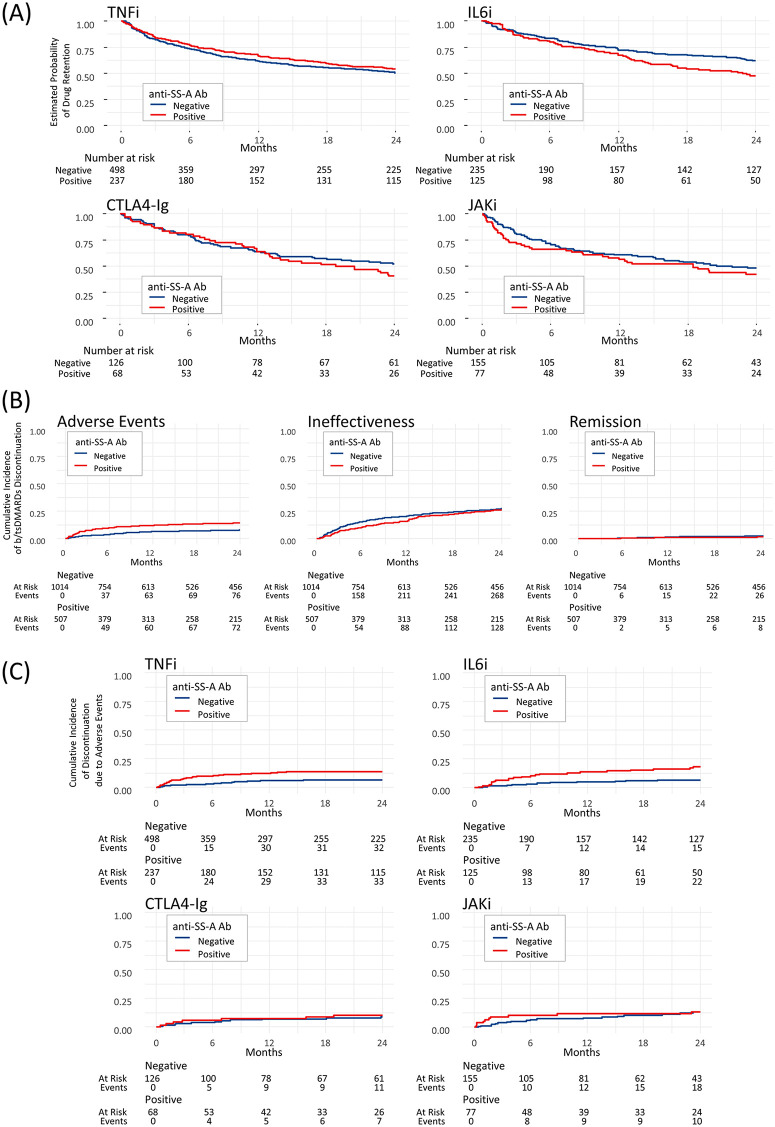
Kaplan–Meier and cumulative incidence curves of b/tsDMARD retention and discontinuation in RA patients with or without anti-SS-A antibodies. **(A)** Kaplan–Meier curves of biologic and targeted synthetic disease-modifying antirheumatic drug (b/tsDMARD) retention by mode of action (MOA: TNF inhibitors [TNFi], IL-6 receptor inhibitors [IL6i], CTLA4-Ig, and JAK inhibitors [JAKi]) in rheumatoid arthritis (RA) patients with or without anti-SS-A antibodies. Anti-SS-A antibody-positive patients tended to show lower retention with IL6i and CTLA4-Ig, while retention appeared similar with TNFi or JAKi. **(B)** Cumulative incidence function curves of b/tsDMARD discontinuation due to adverse events, ineffectiveness, or remission in RA patients with or without anti-SS-A antibodies. Anti-SS-A antibody-positive patients showed a higher cumulative incidence of discontinuation due to adverse events, while no clear differences were observed for ineffectiveness or remission. **(C)** Cumulative incidence function curves of b/tsDMARD discontinuation due to adverse events by MOA (TNFi, IL6i, CTLA4-Ig, JAKi) according to anti-SS-A antibody status. Anti-SS-A antibody positivity was associated with a higher risk of discontinuation due to adverse events with IL6i and TNFi, while no clear differences were observed with CTLA4-Ig or JAKi. The number at risk and the number of events are shown below each plot. The curves are shown for one representative imputed matched dataset (imputation 1). Cumulative incidence functions were estimated in the presence of competing risks. **Abbreviations:** Ab, antibody; b/tsDMARD, biologic and targeted synthetic disease-modifying antirheumatic drug; CI, confidence interval; CTLA4-Ig, cytotoxic T lymphocyte-associated antigen 4-immunoglobulin; IL6i, interleukin-6 receptor inhibitors; JAKi, Janus kinase inhibitors; MOA, mode of action; RA, rheumatoid arthritis; TNFi, tumor necrosis factor inhibitors.

Next, we analyzed the risk of drug discontinuation according to the MOA of b/tsDMARDs (Fig 3A). Among these, in terms of point estimates, anti-SS-A antibody positivity showed a trend toward an increased risk of discontinuation in IL-6 receptor inhibitor and CTLA4-Ig users, whereas the association between anti-SS-A antibody positivity and the risk of discontinuation appeared weaker in TNF inhibitor and JAK inhibitor users; none of these associations reached statistical significance (Table 2A). These findings indicate that IL-6 receptor inhibitors or CTLA4-Ig might reduce the retention rates in anti-SS-A antibody-positive RA patients.

**Table 2:** Hazard ratios (HRs) and subdistribution hazard ratios (sHRs) for b/tsDMARD discontinuation in rheumatoid arthritis patients with or without anti-SS-A antibodies.

A. Overall discontinuation by MOA (Cox HRs)			
**MOA**	**HR**	**95% CI**	**p value**
TNFi	0.86	[0.68–1.10]	0.226
IL6i	1.38	[1.00–1.91]	0.053
CTLA4-Ig	1.40	[0.92–2.11]	0.114
JAKi	1.13	[0.74–1.72]	0.562
**B. Discontinuation by reason (Fine–Gray sHRs)**			
Adverse Events	1.80	[1.28–2.52]	0.000685
Ineffectiveness	0.92	[0.74–1.15]	0.465
Remission	0.68	[0.30–1.56]	0.364
**C. Adverse event–related discontinuation by MOA (Fine–Gray sHRs)**			
TNFi	2.04	[1.22–3.40]	0.0066
IL6i	2.41	[1.24–4.71]	0.0098
CTLA4-Ig	1.17	[0.43–3.15]	0.760
JAKi	1.15	[0.49–2.70]	0.748

Hazard ratios (HRs) and subdistribution hazard ratios (sHRs), with 95% confidence intervals (CIs) and p values, are shown for b/tsDMARD discontinuation in rheumatoid arthritis patients with or without anti-SS-A antibodies after propensity score matching. (2A) Overall discontinuation risk by mode of action (MOA) was estimated using Cox proportional hazards models. (2B) Discontinuation by reason (adverse events, ineffectiveness, and remission) and (2C) adverse event–related discontinuation by MOA were analyzed using Fine–Gray subdistribution hazards models, treating discontinuations due to other reasons as competing events. Effect estimates were obtained within each imputed dataset and pooled across the 100 imputations according to Rubin’s rules (within approach). **Abbreviations**: b/tsDMARD, biologic and targeted synthetic disease-modifying antirheumatic drug; CI, confidence interval; CTLA4-Ig, cytotoxic T lymphocyte-associated antigen 4-immunoglobulin; HR, hazard ratio; IL6i, interleukin-6 receptor inhibitor; JAKi, Janus kinase inhibitor; MOA, mode of action; sHR, subdistribution hazard ratio; TNFi, tumor necrosis factor inhibitor.

### B/tsDMARDs discontinuation by reason

We examined the risk of b/tsDMARD discontinuation according to the reason for treatment termination. For each reason, the cumulative incidence functions of drug discontinuation in the antibody-positive and antibody-negative groups are presented in Fig 3B. Discontinuation due to adverse events was significantly more frequent in the anti-SS-A antibody-positive group (sHR: 1.80, 95% CI: 1.28–2.52; p = 0.000685). In contrast, no significant differences were observed for discontinuation due to ineffectiveness (sHR 0.92, 95% CI 0.74–1.15; p = 0.465) or remission (sHR 0.68, 95% CI 0.30–1.56; p = 0.364) (Table 2B). These findings suggest that anti-SS-A antibody positivity is specifically associated with an increased risk of discontinuation owing to adverse events. Complementary cause-specific Cox analyses yielded consistent findings and are shown in S2 Table. In an exploratory within TNF inhibitor analysis for discontinuation due to ineffectiveness, anti–SS-A antibody positivity was associated with a lower risk in the etanercept/golimumab/ozoralizumab subgroup (including etanercept biosimilars; sHR 0.575, 95% CI 0.35–0.94), whereas the association was not significant in other TNF inhibitors (sHR 0.813, 95% CI 0.51–1.30). The interaction test was not statistically significant (interaction ratio 0.708, 95% CI 0.36–1.39; p for interaction = 0.318) (S3 Table). Descriptive distributions by individual TNF inhibitor are shown in S4 Table.

### MOA-stratified risks of discontinuations due to adverse events

In the MOA-stratified competing-risk analysis restricted to discontinuation due to adverse events, anti-SS-A antibody positivity was associated with a significantly higher risk of discontinuation among patients treated with IL-6 receptor inhibitors (sHR: 2.41, 95% CI: 1.24–4.71, p = 0.0098) or TNF inhibitors (sHR: 2.04, 95% CI: 1.22–3.40, p = 0.0066) compared with antibody-negative patients (Table 2C, Fig 3C). In contrast, no significant difference was observed for CTLA4-Ig (sHR: 1.17, 95% CI: 0.43–3.15, p = 0.760) or JAK inhibitors (sHR: 1.15, 95% CI: 0.49–2.70, p = 0.748). Sensitivity analyses addressing heterogeneity in anti–SS-A assay methods yielded results consistent with the primary analyses for adverse event–related discontinuation.

## Discussion

This study is the first large multicenter investigation to comprehensively evaluate b/tsDMARD retention in anti-SS-A antibody-positive RA patients. The overall retention rate of b/tsDMARDs was similar between the antibody-positive and antibody-negative groups. In MOA-stratified analyses, anti-SS-A antibody positivity showed a trend toward increased discontinuation with IL-6 receptor inhibitors and CTLA4-Ig. Competing-risk analyses revealed that discontinuation due to adverse events was significantly more frequent in anti-SS-A antibody-positive patients, with an especially increased risk observed among those treated with IL-6 receptor inhibitors and TNF inhibitors. These findings highlight that treatment tolerability, rather than overall efficacy, may be a key determinant of drug survival in this subgroup.

Previous reports have assessed the impact of anti-SS-A antibodies in patients receiving specific agents such as TNF inhibitors or abatacept [15,34]. However, few studies have systematically examined treatment retention across drug classes while distinguishing among various causes of discontinuation, including adverse events and ineffectiveness. The present study further demonstrated that the risk of discontinuation due to adverse events may differ by drug class in anti-SS-A antibody-positive patients, providing novel insights into treatment tolerability in this subset.

Our analysis showed a trend toward an increased discontinuation risk among anti-SS-A antibody-positive patients treated with IL-6 receptor inhibitors. Previous studies have reported the favorable efficacy of IL-6 receptor inhibitors in this population [17]. In contrast, our analysis, which focused on reasons for discontinuation, demonstrated greater heterogeneity in treatment response and tolerability. These results suggest that anti-SS-A antibody-positive patients may be more susceptible to adverse effects of IL-6 receptor inhibitors despite their clinical efficacy.

Both the primary competing-risk (Fine–Gray) analysis and complementary cause-specific Cox analysis showed a higher risk of adverse event–related discontinuation in anti–SS-A–positive patients. This increased risk was especially apparent with the use of IL-6 receptor inhibitors and TNF inhibitors. Immune dysregulation and autoantibody production may contribute to the greater frequency of drug-related adverse events in this subgroup. Previous studies have similarly reported that adverse events are a leading cause of TNF inhibitor discontinuation in RA patients [35–38]. Anti-SS-A antibody-positive RA often overlaps with Sjögren disease (SjD), which is characterized by sicca symptoms and extra-articular involvement [39,40]. These features may contribute to the increased susceptibility to adverse events and subsequent treatment discontinuation, particularly with IL-6 receptor inhibitors and TNF inhibitors.

Conversely, we did not observe a significantly increased risk of adverse event-related discontinuation of CTLA4-Ig or JAK inhibitors. Previous studies have suggested favorable efficacy and retention of abatacept in anti-SS-A antibody-positive RA patients [34], and favorable tolerability of these agents in patients with autoimmune overlap [41–44]. The ROSE trial also demonstrated the clinical benefit and tolerability of CTLA4-Ig in RA patients with associated SjD, including those positive for anti-SS-A antibodies [45–47]. Our analysis did not detect an increased risk of discontinuation due to adverse events associated with JAK inhibitors in anti-SS-A antibody-positive patients. However, this finding should be interpreted with caution because few clinical studies have specifically addressed the safety and efficacy of JAK inhibitors in this subgroup. Most available evidence is derived from preclinical studies, which have shown that JAK inhibitors may modulate key pathogenic pathways, such as interferon signaling and autophagy, in both RA and SjD [48]. Further clinical research is needed to establish the safety profile of JAK inhibitors in anti-SS-A antibody-positive RA patients.

From a clinical perspective, these findings suggest that anti-SS-A antibody status may be an important factor to consider when selecting b/tsDMARDs for RA patients. In antibody-positive patients, especially those with comorbidities that may increase susceptibility to adverse events (e.g., sicca symptoms or other extra-articular manifestations), the use of IL-6 receptor inhibitors or TNF inhibitors may require closer monitoring and careful risk–benefit assessment. Conversely, CTLA4-Ig or JAK inhibitors may represent reasonable alternatives in this subgroup, given the absence of a significantly increased risk of adverse event-related discontinuation in our study. Given that treatment decisions are individualized, incorporating serological profiles such as anti-SS-A antibody status into therapeutic planning may help optimize drug retention and minimize discontinuation due to adverse events.

The mean concomitant MTX dose at baseline appeared relatively low; however, this likely reflects the treatment context in Japan [49]. MTX was initially approved for RA in Japan in 1999 at a recommended dose of 6–8 mg/week, and the maximum approved dose (16 mg/week) has been available only since February 2011; recent Japan College of Rheumatology guidance emphasizes stepwise escalation (often targeting 10–12 mg/week) and notes that a substantial proportion of patients do not tolerate 16 mg/week in routine care [50,51]. For example, in a Japanese randomized trial with protocol escalation up to 16 mg/week, the mean MTX dose over 52 weeks was 11.6 mg/week and approximately 30% reached 16 mg/week at Week 52 [51]. Consistently, analyses of Japanese reimbursement data and the IORRA cohort show that MTX dosing was historically constrained (≤8 mg/week in most patients before 2011), with gradual increases in prescriptions >8 mg/week over time [52], and IORRA analyses have evaluated the efficacy and safety of doses >8 mg/week under this background [53]. Importantly, in our cohort, MTX was not used at treatment-course baseline in 57.5% of treatment courses (61.8% of patients), which also contributes to the low mean weekly dose; given that our cohort comprised treatment courses initiating or switching to b/tsDMARDs, this MTX distribution is best interpreted as reflecting real-world practice (e.g., intolerance, contraindications, or prior inadequate response) rather than under-treatment.

The strengths of this study include the use of a large multicenter registry, the simultaneous evaluation of both MOA-stratified and cause-specific drug retention, and the rigorous adjustment for confounders using propensity score matching and multiple imputation. However, as this was a retrospective observational study, residual confounding by unmeasured factors such as the severity of overlapping SjD, antibody titers, or infection risk factors could not be entirely excluded. The reasons for discontinuation were based on clinician assessment rather than standardized criteria. Therefore, the decision to stop or switch between b/tsDMARDs depends on the individual’s clinical judgment. Anti-SS-A antibody titers were not uniformly recorded. In addition, a structured diagnosis of concomitant SjD was not systematically captured in the registry; thus, we could not estimate the prevalence of overlapping SjD among anti–SS-A–positive patients. As supportive information, anti–SS-B and FANA results were available in 71.2% (361/507) and 80.1% (406/507) of anti–SS-A–positive courses (and 69.4% [177/255] and 77.3% [197/255] at the patient level, first course); however, these serologic markers do not substitute for standardized SjD ascertainment and residual confounding may remain. The cohort was limited to facilities with adequate CDAI data, which may have affected the generalizability. The within–TNF inhibitor analysis was exploratory and based on a grouped comparison rather than formal head-to-head comparisons among individual agents; therefore, it may be susceptible to residual confounding, limited event counts, and multiplicity, and should be interpreted cautiously.

## Conclusions

This multicenter study comprehensively evaluated MOA- and cause-specific b/tsDMARD retention in anti-SS-A antibody-positive RA patients. Overall retention rates were similar between antibody-positive and -negative groups. Discontinuation due to adverse events was significantly more common in antibody-positive patients. Among discontinuations due to adverse events, higher risks were observed with IL-6 receptor inhibitors and TNF inhibitors, but not with CTLA4-Ig or JAK inhibitors. These findings suggest that, in anti-SS-A antibody-positive RA, treatment tolerability rather than overall efficacy may be a key factor influencing drug survival, and that anti-SS-A antibody status may help inform therapeutic decision-making.

## Supporting information

S1 Table Baseline characteristics of anti–SS-A antibody-positive and -negative patients before and after propensity score matching. (DOCX)

S2 Table Cause-specific hazard ratios (csHRs) for b/tsDMARD discontinuation by reason in rheumatoid arthritis patients with or without anti–SS-A antibodies (propensity score–matched treatment courses). (DOCX)

S3 Table Exploratory within–tumor necrosis factor inhibitor analysis for discontinuation due to ineffectiveness (Fine–Gray models). (DOCX)

S4 Table Distribution of individual tumor necrosis factor inhibitors and ineffectiveness-related discontinuations in the propensity score–matched cohort (imputation 1). (DOCX)
