# Supplementary material for: Mode of action-specific and cause-specific retention of biologic and targeted synthetic disease-modifying antirheumatic drugs in anti-SS-A antibody-positive rheumatoid arthritis: The ANSWER cohort study
Source: PLoS One. 2026 Mar 18;21(3):e0344747. doi: 10.1371/journal.pone.0344747 (PMC12998854; doi:10.1371/journal.pone.0344747)
Supplement: S1 Table — (DOCX) [file pone.0344747.s001.docx]

**S1 Table. Baseline characteristics of anti–SS-A antibody-positive and -negative patients before and after propensity score matching.**

|  | **Before Matching** | | | | **After Matching** | | | |
| --- | --- | --- | --- | --- | --- | --- | --- | --- |
| **Variable** | **Overall** | **anti-SS-A antibody** | | **Difference** | **Overall** | **anti-SS-A antibody** | | **Difference** |
|  |  | **Negative** | **Positive** |  |  | **Negative** | **Positive** |  |
|  | N = 1,452 | N = 1,197 | N = 255 |  | N = 883 | N = 628 | N = 255 |  |
| Age (years) | 59 ± 16 | 60 ± 16 | 55 ± 15 | 0.30 | 55 ± 16 | 55 ± 17 | 55 ± 15 | 0.03 |
| Sex |  |  |  | 0.54 |  |  |  | 0.04 |
| Male | 281 (19%) | 269 (22%) | 12 (4.7%) |  | 47 (5.3%) | 35 (5.6%) | 12 (4.7%) |  |
| Female | 1,171 (81%) | 928 (78%) | 243 (95%) |  | 836 (95%) | 593 (94%) | 243 (95%) |  |
| Disease Duration  (months) | 92 ± 115 | 91 ± 117 | 98 ± 106 | -0.07 | 93 ± 103 | 90 ± 102 | 98 ± 106 | -0.08 |
| PSL (mg/day) | 1.5 ± 3.9 | 1.5 ± 4.0 | 1.3 ± 2.9 | 0.06 | 1.4 ± 3.4 | 1.4 ± 3.6 | 1.3 ± 2.9 | 0.02 |
| MTX (mg/week) | 3.5 ± 5.1 | 3.5 ± 4.9 | 3.7 ± 5.7 | -0.05 | 3.9 ± 5.3 | 4.0 ± 5.1 | 3.7 ± 5.7 | 0.05 |
| RF positive | 1,040 (74%) | 831 (71%) | 209 (85%) | -0.33 | 731 (83%) | 514 (82%) | 217 (85%) | -0.09 |
| ACPA positive | 1,020 (76%) | 825 (74%) | 195 (84%) | -0.24 | 745 (84%) | 531 (85%) | 214 (84%) | 0.02 |
| Max  b/tsDMARDs Line | 2 ± 2 | 2 ± 2 | 2 ± 2 | -0.18 | 2 ± 2 | 2 ± 2 | 2 ± 2 | -0.05 |
| CDAI | 18 ± 11 | 18 ± 11 | 18 ± 11 | -0.03 | 17 ± 11 | 17 ± 11 | 17 ± 11 | -0.01 |
| Stage |  |  |  | -0.11 |  |  |  | -0.05 |
| 1 | 504 (42%) | 423 (43%) | 81 (38%) |  | 349 (40%) | 250 (40%) | 99 (39%) |  |
| 2 | 290 (24%) | 238 (24%) | 52 (24%) |  | 215 (24%) | 157 (25%) | 58 (23%) |  |
| 3 | 160 (13%) | 127 (13%) | 33 (15%) |  | 123 (14%) | 84 (13%) | 39 (15%) |  |
| 4 | 250 (21%) | 201 (20%) | 49 (23%) |  | 196 (22%) | 137 (22%) | 59 (23%) |  |
| Class |  |  |  | 0.12 |  |  |  | 0.02 |
| 1 | 363 (30%) | 293 (30%) | 70 (32%) |  | 290 (33%) | 208 (33%) | 82 (32%) |  |
| 2 | 620 (51%) | 505 (51%) | 115 (53%) |  | 446 (51%) | 311 (50%) | 135 (53%) |  |
| 3 | 208 (17%) | 180 (18%) | 28 (13%) |  | 138 (16%) | 103 (16%) | 35 (14%) |  |
| 4 | 18 (1.5%) | 15 (1.5%) | 3 (1.4%) |  | 9 (1.0%) | 6 (1.0%) | 3 (1.2%) |  |

Values are presented as mean ± standard deviation (SD) for continuous variables, and n (%) for categorical variables. “Difference” indicates the standardized mean difference (SMD) between anti-SS-A antibody-positive and -negative groups. “Before” and “After matching” refer to datasets prior to and following propensity score matching, respectively. Baseline was defined at the initiation of each patient’s first biologic and targeted synthetic disease-modifying antirheumatic drug (b/tsDMARD) treatment course included in the study. Counts represent the number of patients. Propensity score matching was performed using the following covariates: age, sex, disease duration, glucocorticoid dosage, methotrexate dosage, seropositivity (RF, ACPA), CDAI, radiographic stage, functional class, maximum number of b/tsDMARDs, and prior b/tsDMARDs. Radiographic stage indicates the Steinbrocker stage (1–4), and functional class indicates the global functional class (1–4). For presentation, this supplementary table shows one representative imputed dataset (imputation 1) from the 100 multiply imputed datasets. Similar trends were observed across the other imputed datasets, with all post-matching SMDs < 0.20. **Abbreviations**: ACPA, anti-citrullinated peptide antibody; b/tsDMARD, biologic and targeted synthetic disease-modifying antirheumatic drug; CDAI, Clinical Disease Activity Index; MTX, methotrexate; PSL, prednisolone; RF, rheumatoid factor.
