# Supplementary material for: Mode of action-specific and cause-specific retention of biologic and targeted synthetic disease-modifying antirheumatic drugs in anti-SS-A antibody-positive rheumatoid arthritis: The ANSWER cohort study
Source: PLoS One. 2026 Mar 18;21(3):e0344747. doi: 10.1371/journal.pone.0344747 (PMC12998854; doi:10.1371/journal.pone.0344747)
Supplement: S2 Table — (DOCX) [file pone.0344747.s002.docx]

**S2 Table. Cause-specific hazard ratios (csHRs) for b/tsDMARD discontinuation by reason in rheumatoid arthritis patients with or without anti–SS-A antibodies (propensity score–matched treatment courses).**

| **Cause** | **cause-specific HR** | **95% CI** | **p value** |
| --- | --- | --- | --- |
| Adverse Events | 1.72 | [1.23–2.42] | 0.002 |
| Ineffectiveness | 0.96 | [0.77–1.19] | 0.692 |
| Remission | 0.69 | [0.30–1.57] | 0.382 |

Cause-specific hazard ratios (csHRs), 95% confidence intervals (CIs), and p-values are shown from cause-specific Cox proportional hazards models for each discontinuation reason, treating discontinuations due to other reasons as censoring at the time of discontinuation. Robust variance estimates were used with clustering by matching subclass and patient. Estimates were obtained within each of the 100 multiply imputed datasets and pooled using Rubin’s rules. **Abbreviations**: b/tsDMARD, biologic and targeted synthetic disease-modifying antirheumatic drug; CI, confidence interval; csHR, cause-specific hazard ratio.
