# Supplementary material for: Mode of action-specific and cause-specific retention of biologic and targeted synthetic disease-modifying antirheumatic drugs in anti-SS-A antibody-positive rheumatoid arthritis: The ANSWER cohort study
Source: PLoS One. 2026 Mar 18;21(3):e0344747. doi: 10.1371/journal.pone.0344747 (PMC12998854; doi:10.1371/journal.pone.0344747)
Supplement: S3 Table — (DOCX) [file pone.0344747.s003.docx]

**S3 Table. Exploratory within–tumor necrosis factor inhibitor analysis for discontinuation due to ineffectiveness (Fine–Gray models).**

| **Effect** | **sHR** | **95% CI** | **p-value** |
| --- | --- | --- | --- |
| SSA effect in ETN/GLM/OZR subgroup (sHR) | 0.575 | [0.35-0.94] | 0.026 |
| SSA effect in other TNFi subgroup (sHR) | 0.813 | [0.51-1.30] | 0.385 |
| Interaction (SSA × subgroup) (ratio of sHRs) | 0.708 | [0.36-1.39] | 0.318 |

Subdistribution hazard ratios (sHRs), 95% confidence intervals (CIs), and p-values are shown from Fine–Gray models including an interaction term between anti–SS-A antibody status and TNF inhibitor subgroup (etanercept [including biosimilars], golimumab, and ozoralizumab vs other TNF inhibitors). Estimates were obtained within each of the 100 imputed matched datasets and pooled using Rubin’s rules. **Abbreviations**: CI, confidence interval; ETN, etanercept; GLM, golimumab; OZR, ozoralizumab; sHR, subdistribution hazard ratio; TNFi, tumor necrosis factor inhibitor.
