# Supplementary material for: Mode of action-specific and cause-specific retention of biologic and targeted synthetic disease-modifying antirheumatic drugs in anti-SS-A antibody-positive rheumatoid arthritis: The ANSWER cohort study
Source: PLoS One. 2026 Mar 18;21(3):e0344747. doi: 10.1371/journal.pone.0344747 (PMC12998854; doi:10.1371/journal.pone.0344747)
Supplement: S4 Table — (DOCX) [file pone.0344747.s004.docx]

**S4 Table. Distribution of individual tumor necrosis factor inhibitors and ineffectiveness-related discontinuations in the propensity score–matched cohort (imputation 1).**

| **TNF inhibitor** | **Courses SSA+** | **Courses SSA-** | **Courses total** | **Ineff SSA+** | **Ineff SSA-** | **Ineff total** |
| --- | --- | --- | --- | --- | --- | --- |
| ADA | 31 | 81 | 112 | 10 | 24 | 34 |
| ADA-BS | 1 | 8 | 9 | 0 | 1 | 1 |
| CZP | 27 | 56 | 83 | 6 | 20 | 26 |
| ETN | 65 | 112 | 177 | 11 | 22 | 33 |
| ETN-BS | 12 | 37 | 49 | 1 | 7 | 8 |
| GLM | 55 | 100 | 155 | 11 | 44 | 55 |
| IFX | 43 | 95 | 138 | 10 | 23 | 33 |
| IFX-BS | 1 | 4 | 5 | 0 | 1 | 1 |
| OZR | 2 | 5 | 7 | 1 | 2 | 3 |
| ***ETN/GLM/OZR subgroup**** | 134 | 254 | 388 | 24 | 75 | 99 |
| ***Other TNF inhibitors*** | 103 | 244 | 347 | 26 | 69 | 95 |
| **Overall** | 237 | 498 | 735 | 50 | 144 | 194 |

Values are counts of treatment courses in the propensity score–matched dataset (imputation 1). SSA+ indicates anti–SS-A antibody positivity and SSA−, negativity. Ineff indicates discontinuation due to ineffectiveness. *ETN/GLM/OZR subgroup includes etanercept (including biosimilars), golimumab, and ozoralizumab. **Abbreviations**: ADA, adalimumab; ADA-BS, adalimumab biosimilar; CZP, certolizumab pegol; ETN, etanercept; ETN-BS, etanercept biosimilar; GLM, golimumab; IFX, infliximab; IFX-BS, infliximab biosimilar; OZR, ozoralizumab.
